# Long-Term Lime and Phosphogypsum Amended-Soils Alleviates the Field Drought Effects on Carbon and Antioxidative Metabolism of Maize by Improving Soil Fertility and Root Growth

**DOI:** 10.3389/fpls.2021.650296

**Published:** 2021-07-12

**Authors:** João William Bossolani, Carlos Alexandre Costa Crusciol, Ariani Garcia, Luiz Gustavo Moretti, José Roberto Portugal, Vitor Alves Rodrigues, Mariley de Cássia da Fonseca, Juliano Carlos Calonego, Eduardo Fávero Caires, Telmo Jorge Carneiro Amado, André Rodrigues dos Reis

**Affiliations:** ^1^Department of Crop Science, College of Agricultural Sciences, São Paulo State University, Botucatu, Brazil; ^2^Department of Soil Science and Agricultural Engineering, State University of Ponta Grossa, Ponta Grossa, Brazil; ^3^Soils Department, Center of Rural Sciences, Federal University of Santa Maria, Santa Maria, Brazil; ^4^Department of Biosystems Engineering, School of Sciences and Engineering, São Paulo State University, Tupã, Brazil

**Keywords:** soil fertility, soil amendments, root distribution, Rubisco, sucrose synthase, oxidative stress

## Abstract

Long-term surface application of lime (L) and/or phosphogypsum (PG) in no-till (NT) systems can improve plant growth and physiological and biochemical processes. Although numerous studies have examined the effects of L on biomass and plant growth, comprehensive evaluations of the effects of this practice on net CO_2_ assimilation, antioxidant enzyme activities and sucrose synthesis are lacking. Accordingly, this study examined the effects of long-term surface applications of L and PG on soil fertility and the resulting impacts on root growth, plant nutrition, photosynthesis, carbon and antioxidant metabolism, and grain yield (GY) of maize established in a dry winter region. At the study site, the last soil amendment occurred in 2016, with the following four treatments: control (no soil amendments), L (13 Mg ha^–1^), PG (10 Mg ha^–1^), and L and PG combined (LPG). The long-term effects of surface liming included reduced soil acidity and increased the availability of P, Ca^2+^, and Mg^2+^ throughout the soil profile. Combining L with PG strengthened these effects and also increased SO_4_^2–^-S. Amendment with LPG increased root development at greater depths and improved maize plant nutrition. These combined effects increased the concentrations of photosynthetic pigments and gas exchange even under low water availability. Furthermore, the activities of Rubisco, sucrose synthase and antioxidative enzymes were improved, thereby reducing oxidative stress. These improvements in the physiological performance of maize plants led to higher GY. Overall, the findings support combining soil amendments as an important strategy to increase soil fertility and ensure crop yield in regions where periods of drought occur during the cultivation cycle.

## Graphical Abstract

Schematic summary of the main effects of lime and phosphogypsum on soil fertility, root growth and maize physiology.

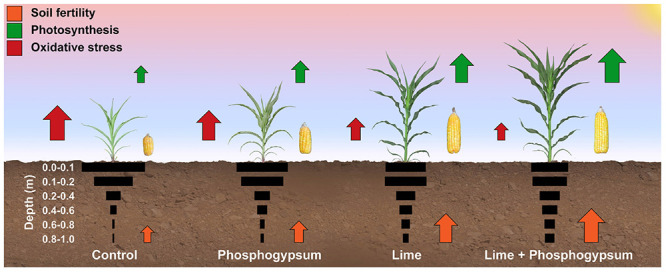

## Introduction

Soil degradation is a serious threat to food security worldwide (Bindraban et al., 2012; FAO, 2015; Gibbs and Salmon, 2015). Global drivers of soil degradation include erosion processes, contamination by heavy metals and other toxic substances, and soil acidification (FAO, 2015). Globally, ∼2.0 billion hectares (ha) of arable soil in the tropics is affected by high acidity (Bian et al., 2013), which is the most important factor limiting crop development and food production capacity in tropical crop systems (Costa and Crusciol, 2016).

Acidic and weathered soils are naturally less fertile due to the limited availability of calcium (Ca^2+^), magnesium (Mg^2+^), phosphorus (P), and high aluminum (Al^3+^) availability, especially in the deepest soil layers (Nora et al., 2017; Costa et al., 2018). Low nutrient availability coupled with high Al^3+^ levels leads to root growth inhibition and, consequently, decreased water and nutrient uptake, resulting in physiological dysfunction and lower yields (Eekhout et al., 2017; Reis et al., 2018). In addition, tropical regions are subject to periods of drought stress, mainly during the autumn/winter seasons (mid-March to September). The combination of low soil fertility and limited root development with prolonged periods of water limitation contributes strongly to lower yields, especially since the vast majority of the cultivated area is in upland conditions (Carmeis Filho et al., 2017). While advances in plant biotechnology have aided the development of plants that are more resistant to abiotic stresses (Gupta et al., 2020), soil management practices that enable crop development even under unfavorable conditions remain fundamental to food production (Gibbons et al., 2014; Holland et al., 2018).

Several management measures have been developed to mitigate the hazardous effects of soil acidification and improve and sustain soil productivity. In particular, liming has become a widespread practice to raise fertility levels and restore soil quality (Holland et al., 2018; Bossolani et al., 2020a, b, 2021a). Liming aims to increase soil pH, which in turn improves effective cation exchange capacity (ECEC) and base saturation (BS) and reduces Al^3+^ and manganese (Mn) concentrations (Crusciol et al., 2019; Bossolani et al., 2020a). However, due its low solubility, the effects of lime occur mostly in the top layers, mainly after surface applications under no-till (NT), and more slowly in deep (Caires et al., 2011). Amendment with phosphogypsum (PG) can also improve soil fertility but cannot correct soil acidity (Zoca and Penn, 2017). PG application increases Ca^2+^ and sulfate (SO_4_^2–^) levels throughout the soil profile, which reduces Al^3+^ toxicity due to AlSO_4_^+^ ion pair formation and decreased Al^3+^ saturation (Caires et al., 2011; Zoca and Penn, 2017). These changes enhance root development in deeper soil layers (Costa et al., 2018), increasing crop tolerance to water limitation (Costa and Crusciol, 2016; Nora et al., 2017). Thus, the surface application of PG is an important complementary strategy to overcome the surface liming limitations (Bossolani et al., 2018, 2020b; Crusciol et al., 2019). Several types of abiotic stress can negatively affect the photosynthetic processes of plants and generate oxidative stress, with deleterious effects on key cellular components and functions (Gómez et al., 2019). Crops established in fertile soils tend to have a greater ability to maintain relatively high levels of growth, stomatal conductance, photosynthesis, and antioxidant metabolism under environmental stresses (e.g., drought) (Kleiner et al., 1992). In addition, less stressed plants exhibit delayed senescence, thereby further increasing photosynthetic capacity and enabling high yields (Gómez et al., 2019). In the present study, a detailed investigation was performed to determine (1) how amending soil with lime and PG under NT influences soil chemical properties and induces improvements in maize root development, nutrient uptake, physiology, and yield and (2) the main changes in the soil and crop nutrition that alter carbon and antioxidant metabolism in maize plants under field conditions.

## Materials and Methods

### Site Description, Experimental Design, and Treatments

The study site was a long-term field experiment (registered on the GLTEN Metadata Portal^1^) with lime and PG application that has been carried out in Botucatu, southeastern São Paulo State, Brazil (22° 83′ 3″ S, 48° 42′ 64″ W, 765 m above sea level), under NT since 2002. The soil was classified as a sandy clay loam kaolinitic and thermic Typic Haplorthox (USDA, 2014). Prior to the beginning of the experiment in 2002, soil granulometric and chemical properties (0.0–0.2 m depth) were determined according to the methods of Kiehl (1979) and van Raij et al. (2001), respectively, as follows: soil pH (0.01 M CaCl_2_ suspension): 4.2; soil organic carbon (SOC): 12.2 g kg^–1^; P (resin): 9.2 mg kg^–1^; exchangeable K^+^: 1.2 mmol_*c*_ kg^–1^; exchangeable Ca^2+^: 14 mmol_*c*_ kg^–1^; exchangeable Mg^2+^: 5 mmol_*c*_ kg^–1^; total acidity at pH 7 (H + Al): 37 mmol_*c*_ kg^–1^, cation exchange capacity (CEC): 57.2 mmol_*c*_ kg^–1^; BS: 35%; aluminum saturation (AS): 65%; sand: 540 g kg^–1^; silt: 110 g kg^–1^; and clay: 350 g kg^–1^. In addition, the clay content at 0.2–0.4 m depth was 360 g kg^–1^. According to Köppen–Geiger’s climate classification (Alvares et al., 2013), the region is a mesothermic type (Cwa) with a humid subtropical climate, dry winters and hot summers. For the period 1956–2019, the average annual maximum and minimum temperatures were 26.1 and 15.3°C, respectively, and the average annual pluvial precipitation was 1,360 mm (Unicamp, 2020).

The experimental design was a randomized complete block with four treatments and four replicates. The treatments were composed by: (i) control (16 years without soil amendments with intensive crop cultivation and fertilizer inputs); (ii) exclusive application of PG; (iii) exclusive application of lime (L); and (iv) combined application of L and PG (LPG). Liming occurred by application of sedimentary dolomitic lime [CaMg(CO_3_)_2_]^2^, with 233 g kg^–1^ CaO and 175 g kg MgO. The PG contained 280 g kg^–1^ CaO, 150 g kg^–1^ S, <1 g kg^–1^ P, and <1 g kg^–1^ fluorine (F). The soil amendments were applied in 2002, 2004, 2010, and 2016 based on the results of annual sampling. The criterion for reapplying the treatments was BS ≤50% in the L treatment. The lime rate was calculated to increase the BS in the topsoil (0.0–0.2 m) to 70% according to the criterion proposed by van Raij et al. (1997) and was 2.7 Mg ha^–1^ in 2002 and 2.0 Mg ha^–1^ in 2004 and 2010. In October 2016, a new application was necessary, however, the lime rate calculation method used was revised considering the layer of 0.0–0.4 m depth, resulting in 13 Mg ha^–1^ of L, a rate used to rise the BS to 70%. When lime reapplication was required, PG was also reapplied. In 2002, 2004, and 2010, the rates of PG application were determined according to van Raij et al. (1997) by multiplying the clay content in the 0.2–0.4 m layer by a factor of 6, resulting in a rate of 2.1 Mg ha^–1^. In 2016, the most recent methodology for PG application in tropical soils proposed by Caires and Guimarães (2018), which is intended to increase Ca^2+^ saturation in the ECEC to 60% in the 0.2–0.4 m soil layer, was used, resulting in a higher PG rate of 10 Mg ha^–1^. After the last soil amendments reapplication, a micronutrient based fertilizer was applied over a total area at rates of 3 kg ha^–1^ B + 1 kg ha^–1^ Cu + 1 kg ha^–1^ Mn + 10 kg ha^–1^ Zn + 0.2 kg ha^–1^ Mo, in order to avoid unavailability of micronutrients due to the increase in pH by liming.

This study characterizes the long-term residual effects of these treatments in the first and second years after the last soil amendment reapplications in 2016. Several crops were grown during the agricultural years from 2002 to 2018. Previous crops grown and details of the L and PG applications are shown in Supplementary Table 1. Except for the first applications of lime and PG in 2002, when the experiment under NT began, all applications were applied to the soil surface.

### Crop Management

Maize (simple hybrid P3707VYH; 60,000 plants ha^–1^; DuPont Pioneer^®^, Johnston, IA, United States) was sown in March 2017 and 2018. For both growing seasons, fertilization was performed at sowing with 28 kg ha^–1^ N, 98 kg ha^–1^ P_2_O_5_, and 56 kg ha^–1^ K_2_O. In addition, a topdressing fertilization with 90 kg N ha^–1^ (NH_4_)_2_SO_4_ (Cantarella et al., 1997) as ammonium sulfate was performed at the V_4_ phenological stage (Ritchie et al., 1993). The field plots consisted of 14 rows with a length of 9 m spaced 0.45 m apart (56.7 m^2^). The plots were spaced 8 m from each other to avoid cross-contamination from surface runoff containing fertilizers as a consequence of heavy rainfall or during treatment applications and sowing.

### Meteorological Data

**FIGURE 1:**
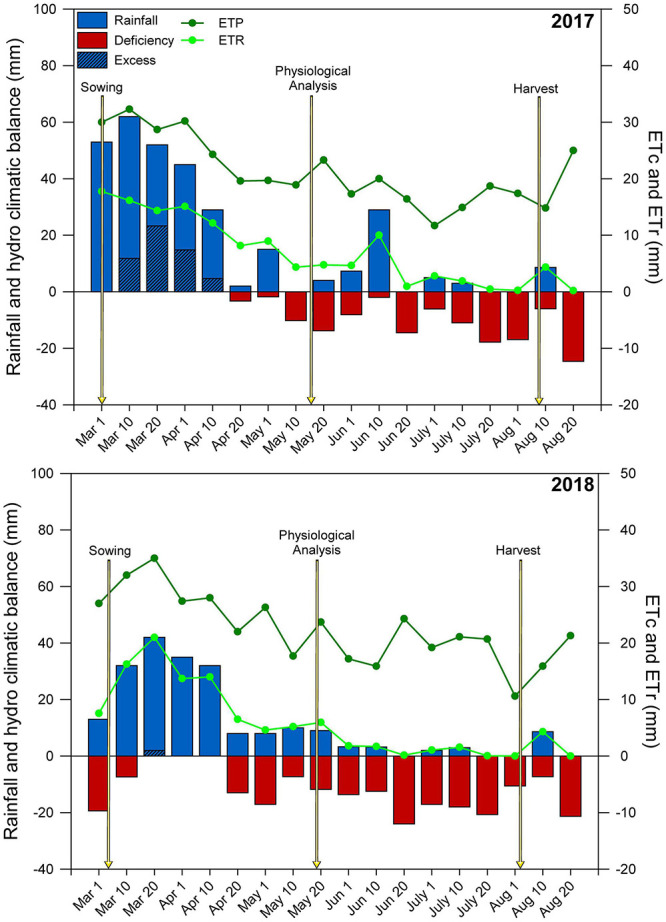
Climatological water balance at Botucatu-SP, Brazil, during the maize crop cycle. ETc, crop evapotranspiration; ETr, real evapotranspiration. The arrows indicate the managing and sampling time.

Throughout the experimental period, meteorological data (rainfall, solar radiation, wind speed, relative humidity, and maximum and minimum temperatures) were obtained through automatic meteorology station installed close to the experimental area. The evapotranspiration reference (ET_0_) was calculated using the Penman-Monteith method (Allen et al., 1998). The crop evapotranspiration (ETc) was calculated using the crop coefficient (Kc) for each stage of the crop’s phenological stage (Allen et al., 1998). Using the rainfall data, the climatological water balance was monitored and calculated using electronic spreadsheets (Rolim et al., 1998), following the procedure of Thornthwaite and Mather (1955) to determine the real evapotranspiration (ETr). The climatological water balance of the two growing seasons is shown in Figure 1.

### Soil Chemical Properties Analysis

In September 2018 (24 months after the last reapplication of soil amendments), eight individual soil subsamples were randomly taken at depths of 0.0–0.1, 0.1–0.2, 0.2–0.4, 0.4–0.6, 0.6–0.8, and 0.8–1.0 m in each plot, except for SOC, P, Fe, Mn, Cu, and Zn, that were sampled at a depth of 0.0–0.2 m. The samples were dried, sieved (2 mm mesh) and analyzed according to van Raij et al. (2001). The soil chemical analysis included soil pH (0.01 M CaCl_2_), SOC (Walkley and Black, 1934), exchangeable cations (K^+^, Ca^2+^, and Mg^2+^) and P extracted by ion-exchange resin and determined by atomic absorption spectrophotometry (AAS) and colorimetric method (van Raij et al., 2001), respectively. Total acidity (H + Al) was estimated using Smith-McLean-Pratt solution (Shoemaker et al., 1961), whereas exchangeable Al^3+^ was extracted using 1 M KCl and both determined by titration with 0.025 M NaOH solution. SO_4_^2–^-S content was extracted by 0.01 M calcium phosphate solution (Bardsley and Lancaster, 1960), and determined by a turbidimetric method. Cationic micronutrients (Fe, Mn, Cu, and Zn) were extracted with a solution containing 0.005 M diethylenetriaminepentaacetic acid (DTPA) pH 7.3, 0.1 M triethanolamine (TEA) and 0.01 M CaCl_2_ and determined by AAS (van Raij et al., 2001).

### Root Sampling and Dry Matter Determination

In both growing seasons, at maize full flowering (Ritchie et al., 1993), eight root subsamples (four subsamples from the plant rows and four subsamples from the middle of the inter-rows) were collected randomly from each plot and combined. A galvanized steel probe with a 82-mm-diameter cutting tip was used at depths of 0.0–0.1, 0.1–0.2, 0.2–0.4, 0.4–0.6, 0.6–0.8, and 0.8–1.0 m. Roots were carefully separated from soil and other residues by washing under a flow of swirling water over a 0.5-mm mesh sieve. The samples were dried in a forced-air oven at 60°C for 72 h to measure root dry matter, expressed in g m^–3^ and subsequently estimated to Mg ha^–1^ in the 0.0–1.0 m layer. The root dry matter distribution was calculated from the ratio of root dry matter in each layer to total root dry matter.

### Leaves Sampling for Crop Nutrition and Physiologic Analysis

Nutritional, physiological, and biochemical analyses occurred on the same diagnostic leaves, when maize plants were at the full flowering (R_2_ phenological stage) (Ritchie et al., 1993). The diagnostic leaves selected were the fully expanded leaves in the top third of the maize canopy. Leaves sampling occurred immediately after the gas exchange analyses. Nutritional analysis occurred in dried and milled plant material, whereas the leaves for physiological analysis were placed in liquid nitrogen and stored at −80°C until further analysis.

#### Nutritional Determination

Leaf N was extracted by sulfuric digestion and determined by Kjeldahl method. Phosphorus, K, Ca, Mg, sulfur (S), Fe, Mn, Cu, and Zn extraction occurred by nitroperchloric digestion, and determined by AAS. Both methods are described by AOAC (2016).

#### Gas Exchange Parameters

Gas exchange assessments consisted of non-destructive analyses of fifteen diagnostic maize leaves using a using a Portable Infrared Gas Analyzer CIRAS-3 Portable Photosynthesis System (PP Systems Inc., Amesbury, MA, United States). The following parameters were determined on the diagnostic leaves of maize: net photosynthetic rate expressed as area (*A*; μmol CO_2_ m^–2^ s^–1^); stomatal conductance (*gs*; mol H_2_O m^–2^ s^–1^); internal CO_2_ concentration in the substomatal chamber (*ic*; mmol CO_2_ mol^–1^ air); leaf transpiration (*E*; mmol H_2_O m^–2^ s^–1^); and water use efficiency [WUE; μmol CO_2_ (mmol H_2_O)^–1^], calculated by the *A*/*E* ratio. Readings began after the air temperature in the chamber was adjusted to 28°C, with 380 ppm CO_2_ and 1,000 μmol m^–2^ s^–1^ of photosynthetically active radiation (PAR) supplied by LED lamps. The measurements were performed between 8:00 and 10:00 a.m. The minimum equilibration time before performing the reading was 3 min.

#### Photosynthetic Pigments

To determine photosynthetic pigments (chlorophyll *a*, *b*, total chlorophyll, and total carotenoids), five discs were cut between the edge and central rib of maize leaves using a paper punch (0.5 cm in diameter). The discs were stored for 24 h in capped glass vials wrapped in aluminum foil and containing 2 mL of N, N-dimethylformamide (DMF) according to the methodology proposed by Lichtenthaler (1987). The pigment concentrations were quantified in a spectrophotometer at wavelengths of 664, 647, and 480 nm for chlorophyll *a*, *b* and carotenoids, respectively. The absorbance was read after mixing 1 mL of the extract with 1 mL of distilled water. The calculations for the pigment concentrations were in accordance with the methods proposed by Wellburn (1994).

#### Ribulose-1,5-Bisphosphate Carboxylase/Oxygenase Activity (Rubisco, EC 4.1.1.39)

Total Rubisco activity (determined only in the second growing season) was measured according to the method described by Reid et al. (1997). Frozen plant material (0.3 g) was ground with a mortar and pestle under liquid nitrogen and suspended in extraction buffer containing 1.5 mL of 58 mM potassium phosphate and 1 mM ethylenediaminetetraacetic acid (EDTA). The homogenized material was centrifuged at 14,000 rpm for 25 min at 4°C, and the supernatant was stored at 4°C (adapted from Sage et al., 1988; Reid et al., 1997). The Rubisco incubation buffer contained 100 mM bicine-NaOH pH 8.0, 25 mM potassium bicarbonate (KHCO_3_), 20 mM magnesium chloride (MgCl_2_), 3.5 mM ATP, 5 mM phosphocreatine, 0.25 mM NADH, 80 nkat glyceraldehyde-3-phosphate dehydrogenase, 80 nkat 3-phosphoglyceric phosphokinase, and 80 nkat creatine phosphokinase. A 70-μL aliquot of the supernatant was incubated with 900 μl of the incubation buffer at 30°C for 5 min in the absence of ribulose-1,5-bisphosphate (RuBP) to enable carbamylation of Rubisco. NADP oxidation was initiated by adding 30 μL of 16.66 mM RuBP directly into the cuvette. Readings were obtained in a spectrophotometer at a wavelength of 340 nm. Rubisco activity was calculated from the difference in the absorbance readings at 0 and 1 min (without removing the cuvette from the spectrophotometer) and expressed in μmol min^–1^ mg protein^–1^.

#### Sucrose Synthase Activity (EC 2.4.1.13)

To extract sucrose synthase (Susy) (determined only in the second growing season), 0.5 g of frozen plant material was ground with a mortar and pestle under liquid nitrogen and suspended in extraction buffer containing 50 mM HEPES buffer pH 7.0, 2 mM MgCl_2_, 2 mM dithiothreitol (DTT) and 1 mM EDTA (Dejardin et al., 1997). The homogenized material was centrifuged at 14,000 rpm for 20 min at 4°C, and the supernatant was stored at 4°C. The incubation buffer consisted of 0.1 M MES buffer pH 6.0, 5 mM MgCl_2_, 0.3 M sucrose and 5 mM uridine 5′-trihydrogen diphosphate (UDP). A 0.5-mL aliquot of the supernatant was incubated with 3.5 mL of incubation buffer at 37°C for 30 min, and the reaction was terminated by adding 100 μL of potassium hydroxide (30% KOH; w/v) and heating at 100°C for 5 min. Readings were obtained in a spectrophotometer at a wavelength of 540 nm, and the results were expressed as μmol sucrose g^–1^ fresh weight (FW) h^–1^.

#### Sucrose Concentration

The sucrose concentration was determined from 1 g of frozen plant material extracted in 10 mL of MCW solution [60% methanol, 25% chloroform, and 15% water; (w/v)] after maceration by a mortar and pestle. The homogenized material was centrifuged at 8,000 rpm for 10 min at 4°C. An aliquot of 4 mL of the supernatant was removed and mixed with 1 mL of chloroform and 1.5 mL of distilled water in another tube. After separation of the phases, the sucrose content was determined in the aqueous phase as described by Bieleski and Turner (1966). In brief, a 50-μL aliquot of the aqueous phase was mixed with 500 μL of 30% KOH (w/v) and 2 mL of H_2_SO_4_. After homogenization by a vortex mixer, the tube was heated at 100°C for 10 min. After cooling, readings were obtained in a spectrophotometer at a wavelength of 490 nm. The sucrose concentration was determined by reference to a standard sucrose curve and expressed as mg g^–1^ FW.

#### Hydrogen Peroxide and Lipid Peroxidation

The hydrogen peroxide (H_2_O_2_) concentration was determined according to the methodology proposed by Alexieva et al. (2001). Frozen plant material (0.4 g) was ground with a mortar and pestle under liquid nitrogen, and 4 mL of 0.1% trichloroacetic acid (TCA) was added as extraction buffer. The homogenized material was centrifuged at 12,000 rpm for 15 min at 4°C. An aliquot of 200 μL of supernatant was mixed with 200 μL of 100 mM potassium phosphate buffer (pH 7.5) and 800 μL of 1 M potassium iodide and incubated at 1°C for 1 h. After warming to room temperature, readings were obtained in a spectrophotometer at a wavelength of 390 nm. The leaf concentration of H_2_O_2_ was determined by reference to a standard curve and expressed as μmol g^–1^ FW. Lipid peroxidation, or malondialdehyde (MDA), was measured according to the methodology proposed by Heath and Packer (1968). The extraction was carried out using 0.4 g of frozen plant material ground with a mortar and pestle under liquid nitrogen and suspended in 4 mL of 0.1% (w/v) TCA + 20% (w/v) polyvinylpolypyrrolidone (PVPP). The homogenized material was centrifuged at 10,000 rpm for 15 min at 4°C. A 250-μL aliquot of supernatant was mixed with 1 mL of 20% TCA + 0.5% thiobarbituric acid (w/v) solution to start the reaction. The reactions were heated at 95°C for 30 min and then placed on ice for 10 min. The reactions were centrifuged for 10 min at 10,000 rpm, and readings of the supernatant were obtained in a spectrophotometer at wavelengths of 535 and 600 nm. The results were expressed as nmol MDA g^–1^ FW.

#### Soluble Protein

Proteins were extracted from 1.5 g of frozen plant material ground with a mortar and pestle under liquid nitrogen and suspended in 20% PVPP and extraction buffer containing 100 mM potassium phosphate pH 7.5, 1 mM EDTA, and 1 mM DDT. The homogenized material was centrifuged at 10,000 rpm for 25 min at 4°C, and the supernatant was stored in Eppendorf tubes in a freezer at −80°C. The soluble protein concentration was determined using BSA (bovine serum albumin) as a standard according to the method proposed by Bradford (1976). Aliquots of 100 μL of the protein extract were mixed with 5 mL of Bradford reagent and analyzed in a spectrophotometer at a wavelength of 595 nm. The results and protein extract were used to determinate the activities of superoxide dismutase (SOD), catalase (CAT), ascorbate peroxidase (APX), and glutathione reductase (GR).

#### Superoxide Dismutase (EC:1.15.1.1)

Superoxide dismutase activity was determined according to Giannopolitis and Ries (1977). The reaction was conducted in a reaction chamber under illumination with a 15-W fluorescent lightbulb at 25°C after adding 2 mL of 50 mM potassium phosphate buffer pH 7.8, 250 μL of 13 mM methionine, 200 μL of 75 mM nitroblue tetrazolium (NBT), 200 μL of 0.1 mM EDTA and 250 μL of 2 μM riboflavin to 50 μL of protein extract. The tubes were vortexed and placed inside the chamber (total absence of ambient light) for 15 min top permit the formation of the blue formazan compound by photoreaction of NBT. A control was prepared for each sample using the same conditions, except that the tubes were covered with aluminum foil to prevent light exposure. After 15 min, the reactions were vortexed, and readings were obtained in a spectrophotometer at a wavelength of 560 nm. The results were expressed as U (unit) SOD mg^–1^ protein.

#### Catalase (1.11.1.6)

Catalase activity was determined by monitoring the degradation of H_2_O_2_ according to the methodology proposed by Azevedo et al. (1998). A 25-μL aliquot of protein extract was added to a mixture containing 1 mL of 100 mM potassium phosphate buffer pH 7.5 and 2 μL of 30% H_2_O_2_ (v/v) in a test tube and mixed quickly by vortexing. Enzyme activity was determined by the decomposition of H_2_O_2_ during a 2-min interval as measured using a spectrophotometer at a wavelength of 240 nm. The results were expressed as μmol min^–1^ mg^–1^ protein.

#### Ascorbate Peroxidase (EC:1.11.1.11)

Ascorbate peroxidase activity was determined according to Gratão et al. (2008). A 100-μL aliquot of protein extract was added to 600 μL of 80 mM potassium phosphate buffer pH 7.0, 100 μL of 5 mM ascorbate and 100 μL of 1 mM EDTA and homogenized by vortexing. The mixture was incubated for 5 min at 30°C in the dark. A 100-μL aliquot of 1 mM H_2_O_2_ was then added, and readings were obtained immediately in a spectrophotometer at a wavelength of 290 nm for 2 min. The results were expressed as μmol min^–1^ mg^–1^ protein.

#### Glutathione Reductase (EC 1.6.4.2)

Glutathione reductase activity was determined according to the methodology described by Gomes-Junior et al. (2006). One milliliter of 100 mM potassium phosphate buffer pH 7.5, 500 μL of 1 mM nitrobenzoic acid (DTNB), 100 μL of 1 mM oxidized glutathione (GSSG) and 100 μL of 0.1 mM NADPH were added to an Eppendorf tube and mixed thoroughly. A 50-μL aliquot of the supernatant was added and vortexed. The solution was then transferred to a cuvette, and the absorbance at 412 nm was immediately recorded for 2 min. The results were expressed as μmol min^–1^ mg^–1^ protein.

### Shoot Dry Matter and Grain Yield of Maize

Maize shoot dry matter (SDM) and grain yield (GY) were evaluated at the harvest stage based on the relationship between the masses (shoot and grain) obtained in the interior (5.4 m^2^) of each plot and their respective water contents (0 and 130 g kg^–1^ of water for SDM and GY, respectively).

### Statistical Analysis

Means were subjected to tests of homoscedasticity followed by the Anderson-Darling test of normality (Nelson, 1998). To evaluate homogeneity, Levene’s test in the Minitab statistical program was used. Subsequently, the means were subjected to analysis of individual variance (ANOVA) by the *F*-test (*p* ≤ 0.05) and, when significant, analyzed using the modified *t*-test [Fisher’s protected least significant difference (LSD) at *p* ≤ 0.05]. Redundancy analysis (RDA) was performed to determine the correlations among soil fertility × crop nutrition (average of two growing seasons), soil fertility × crop physiology (average of two growing seasons), and crop nutrition × crop physiology. The Monte Carlo permutation test was applied with 999 random permutations to verify the significance of soil chemical properties, crop nutrition, and for physiological responses. One-way PERMANOVA (Anderson, 2005) was used to group treatments by similarity. Heatmaps were constructed by calculating the Pearson’s correlation coefficients (*p* ≤ 0.05), and only significant correlations are shown.

## Results

### Weather Conditions

In the first and second growing seasons, maize received 315 and 210 mm of pluvial precipitation, respectively (Figure 1). From early-March (maize sowing) until early July (maize physiological maturity) of each year, only small amounts of rain occurred, resulting in a negative hydric balance.

### Soil Fertility and Root Development

**FIGURE 2:**
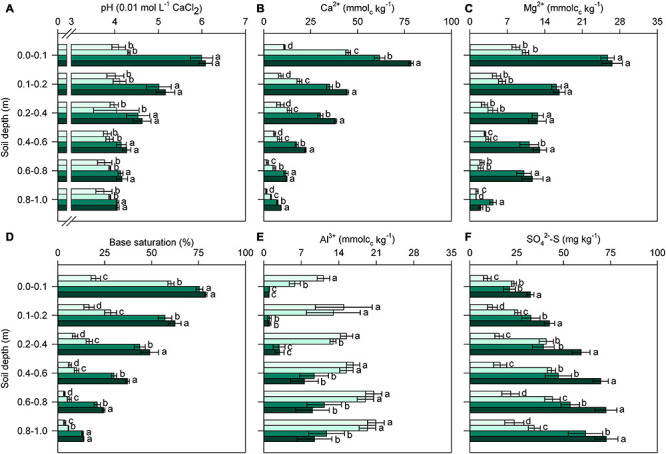
Changes in soil pH **(A)**, exchangeable calcium (Ca^2+^) **(B)** and magnesium (Mg^2+^) **(C)**, base saturation (BS) **(D)**, exchangeable aluminum (Al^3+^) **(E)**, and sulfate (SO_4_^2–^-S) **(F)** in the soil profile as affected by surface-applied lime (L), phosphogypsum (PG), and lime + phosphogypsum (LPG) treatments. Different lower-case letters for each soil depth indicate significant differences between treatments by Student’s *t*-test at *p* ≤ 0.05. Error bars express the standard error of the mean (*n* = 4).

Twenty-four months after the last reapplication of soil amendments, significant variations (*p* < 0.01; Supplementary Table 2) in soil chemical properties among the treatments were observed in all layers of the soil profile (Figure 2). Surface amendment with L (regardless of PG addition) increased the soil pH in all soil layers (0.0–1.0 m); additionally, acidity neutralization was not improved by PG application compared with the control (Figure 2A). By contrast, significant increases in Ca^2+^ availability occurred in all soil layers when soil was amended with L and, in particular, LPG (Figure 2B). PG increased the levels of Ca^2+^ in topsoil in relation to the control, resulting in high BS values (Figure 2D), but BS remained lower in the PG treatment than in the L and LPG treatments. Mg^2+^ availability did not differ between L and LPG, but was higher in both of these treatments than in the control and PG treatments (Figure 2C). BS was slightly improved by LPG compared with L alone, mainly in deeper soil layers (0.1–0.8 m) (Figure 2D).

**FIGURE 3:**
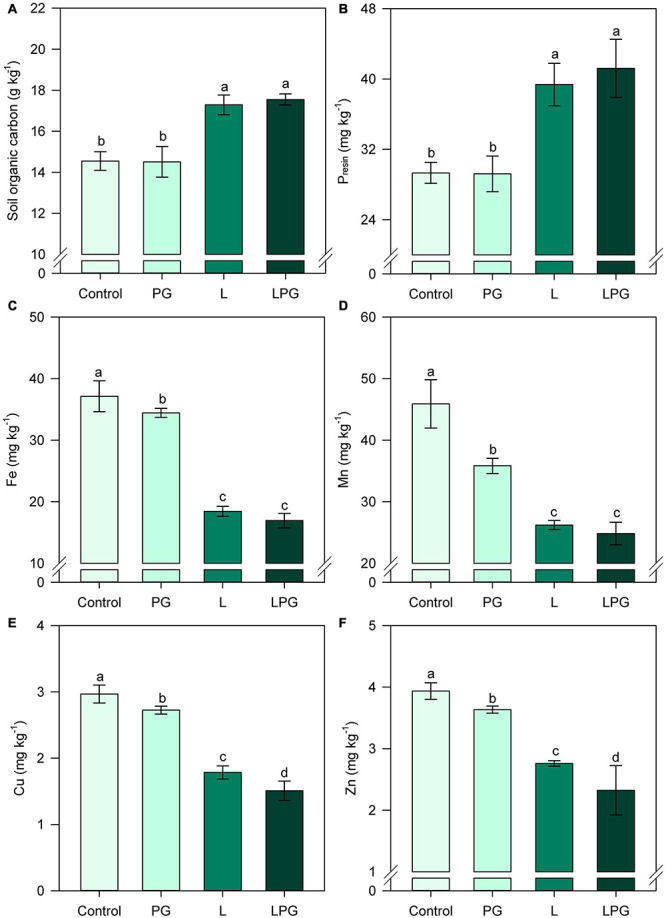
Changes in soil organic carbon (SOC) **(A)**, phosphorus (P) **(B)**, iron (Fe) **(C)**, manganese (Mn) **(D)**, copper (Cu) **(E)**, and zinc (Zn) **(F)** at 0.0–0.2 m depth as affected by surface-applied lime (L), phosphogypsum (PG), and lime + phosphogypsum (LPG) treatments. Different lower-case letters for each soil depth indicate significant differences between treatments by Student’s *t*-test at *p* ≤ 0.05. Error bars express the standard error of the mean (*n* = 4).

Aluminum availability was strongly reduced by the application of L and LPG, mainly in the uppermost layers (0.0–0.4 m depth); in soil depths below 0.4 m, there was no difference in aluminum availability between L and LPG (Figure 2E). In addition, the application of PG alone did not reduce levels of exchangeable Al^3+^ in layers below 0.4 m compared with the control treatment. The SO_4_^2–^-S concentration was higher throughout the soil profile when LPG was applied; in the treatments with L or PG alone, the SO_4_^2–^-S concentrations were similar and higher than that in the control treatment (Figure 2F). SOC, P, Fe, Mn, and Zn contents in the topsoil (0.0–0.2 m) were significantly altered (*p* < 0.01; Supplementary Table 3) by soil amendments (Figure 3). In general, L-amended soils (regardless of PG addition) provided the highest levels of SOC (Figure 3A) and P (Figure 3B) and the lowest concentration of Fe, Mn, Cu, and Zn (Figure 3).

**FIGURE 4:**
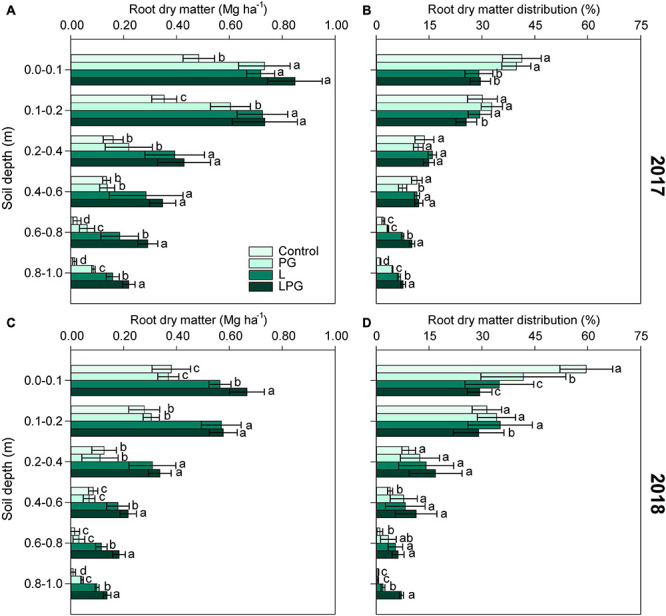
Root dry matter **(A,C)** and root dry matter distribution **(B,D)** in the soil profile as affected by surface-applied lime (L), phosphogypsum (PG), and lime + phosphogypsum (LPG) treatments. Different lower-case letters for each soil depth indicate significant differences between treatments for each growing season by Student’s *t*-test at *p* ≤ 0.05. Error bars express the standard error of the mean (*n* = 4).

Maize root development composed by dry matter production and distribution, changed (*p* < 0.01; Supplementary Table 4) by the long-term surface application of soil amendments (Figure 4). In both growing seasons, root dry matter increased in L-amended soil, but combining L to PG (LPG treatments), the effects were enhanced, especially at deeper layers (below 0.4 m depth) (Figures 4A,C). Between treatments became more evident as the soil depth increased. Regardless of growing season, the root system distribution in the soil profile revealed that the density of roots was highest at 0.0–0.2 m soil layer in the control and PG treatments, with a lower proportion of roots in layers deeper than 0.2 m. In L-amended and, in particular, LPG-amended soil, the root distribution was more uniform throughout the soil profile (Figures 4B,D). Treatment with PG alone improved root development and distribution compared with the control treatment, but the effects of PG alone were smaller than those of the L and LPG treatments. LPG-treatment provided the highest distribution of maize roots in the deepest soil depth.

### Plant Nutrition

Leaf macronutrient concentrations increased (*p* < 0.05; Supplementary Table 5) in response to L and, in particular, LPG in both growing seasons (Table 1). With the exception of K which increased only in 2018, higher leaf concentration of N, P, Ca, Mg, and S occurred in both growing seasons in these treatments compared with the control and PG treatments. By contrast, the concentrations of Fe, Mn, and Zn reduced in treatments L-based (L and LPG), as occurred according to the soil chemical analysis (Figure 3). Among the micronutrients, leaf Cu concentration presented the smallest variation among micronutrients, showing changes only in 2018, where the control treatment provided higher concentrations than the other treatments.

**TABLE 1:** Influence of surface-applied lime (L), phosphogypsum (PG), and lime + phosphogypsum (LPG) on nutrient (N, P, K, Ca, Mg, S, Fe, Mn, Cu, and Zn) concentrations in the leaves of maize cultivated in two growing seasons in a long-term no-till system.

**Leaf nutrients**	**Units**	**Control**		**PG**		**L**		**LPG**	
		**2017**	**2018**	**2017**	**2018**	**2017**	**2018**	**2017**	**2018**
N	g kg^–1^	27.9 b	22.9 c	30.9 b	25.1 c	38.6 a	29.4 b	39.9 a	36.8 a
P	g kg^–1^	2.21 b	1.81 b	2.32 ab	1.99 ab	2.33 ab	2.01 ab	2.41 a	2.21 a
K	g kg^–1^	20.6 a	16.1 b	20.1 a	17.7 ab	20.8 a	18.3 ab	21.8 a	20.2 a
Ca	g kg^–1^	1.81 c	2.13 b	4.18 a	2.34 ab	2.93 b	2.62 ab	3.78 ab	2.77 a
Mg	g kg^–1^	2.20 b	1.81 c	2.03 b	1.99 c	5.30 a	4.39 b	5.88 a	5.63 a
S	g kg^–1^	1.32 d	1.08 b	2.11 c	1.19 b	3.07 b	2.82 a	3.96 a	3.01 a
Fe	mg kg^–1^	276 a	226 a	235 a	249 a	149 b	157 b	222 ab	164 b
Mn	mg kg^–1^	30.6 a	25.1 a	23.8 b	27.6 a	21.5 b	19.6 b	21.9 b	19.4 b
Cu	mg kg^–1^	13.7 a	11.7 ab	11.5 a	12.9 a	12.1 a	9.82 b	13.2 a	13.0 a
Zn	mg kg^–1^	78.4 a	64.3 a	82.1 a	70.6 a	42.4 b	39.4 b	46.5 b	35.9 b

*Different lower-case letters on the lines indicate significant differences between treatments by Student’s *t*-test at *p* ≤ 0.05.*

*Nitrogen (N), phosphorus (P), potassium (K), calcium (Ca), magnesium (Mg), sulfur (S), iron (Fe), manganese (Mn), cupper (Cu), and zinc (Zn).*

### Photosynthetic Pigments and Gas Exchange Measurements

**FIGURE 5:**
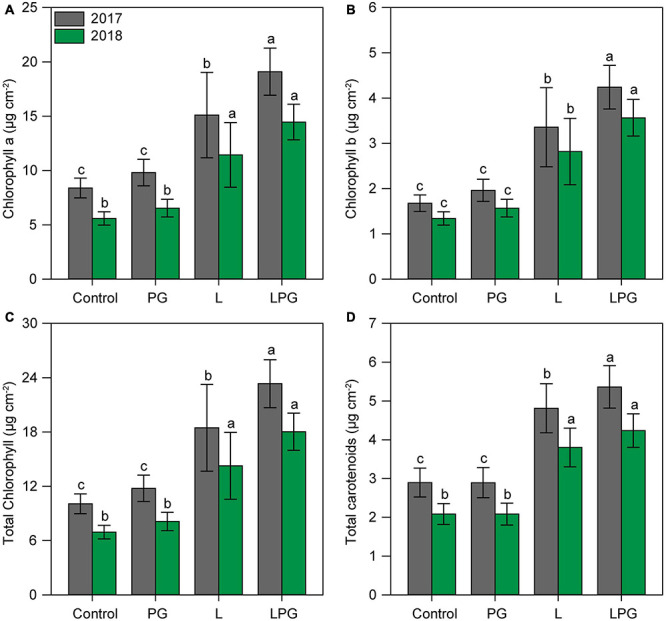
Chlorophyll *a* **(A)**, chlorophyll *b* **(B)**, total chlorophyll **(C)**, and carotenoids **(D)** contents in maize leaves as affected by surface-applied lime (L), phosphogypsum (PG), and lime + phosphogypsum (LPG) treatments. Different lower-case letters indicate significant differences between treatments for each growing season by Student’s *t*-test at *p* ≤ 0.05. Error bars express the standard error of the mean (*n* = 4).

**FIGURE 6:**
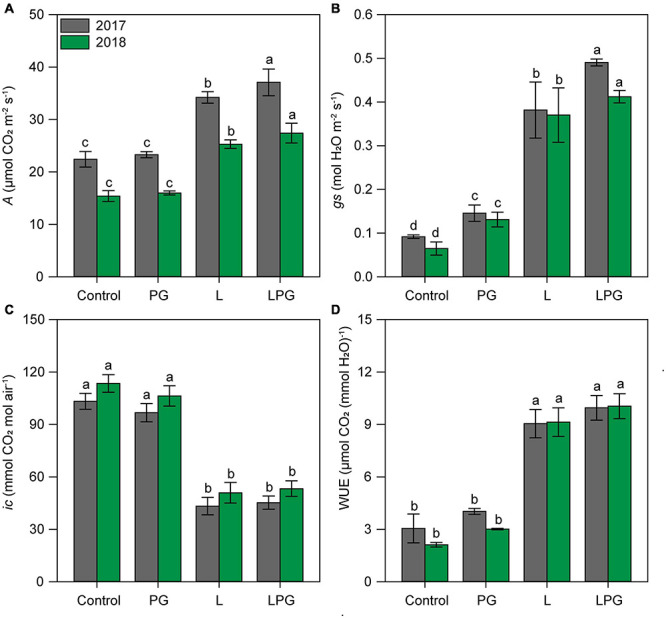
Net photosynthesis rate–*A* **(A)**, stomatal conductance–*gs* **(B)**, internal CO_2_ concentration–*ic* **(C)**, and water use efficiency–WUE **(D)** in maize leaves as affected by surface-applied lime (L), phosphogypsum (PG), and lime + phosphogypsum (LPG) treatments. Different lower-case letters indicate significant differences between treatments for each growing season by Student’s *t*-test at *p* ≤ 0.05. Error bars express the standard error of the mean (*n* = 4).

Regardless of growing season, the application of L and particularly LPG increased (*p* < 0.01; Supplementary Table 6) the concentrations of chlorophylls (*a*, *b*, and total) and carotenoids compared with the control and PG treatments (Figure 5). On average, considering all photosynthetic pigments, LPG-amended soil provided maize plants with 137% more pigments than the control treatment, followed by 90% more in the treatment with L and 15% with PG. Leaf gas exchange of maize in both growing seasons improved by applying LPG, followed by L (Figure 6) compared with control and PG. In LPG-amended soil, maize plants presented the largest *A* rate compared with control treatments (LPG = 70%; L = 57%), as well as *gs* rates increased by 478% in LPG, by 380% in L, and by 76% in PG treatments (Figures 6A,B). Lowest rates of *ic* (Figure 6C) and *E* (Supplementary Figure 1) also occurred in these treatments (L and LPG), although differences did not occur between them. As a consequence of improvements in *A* and *E* rates, higher WUE occurred maize cultivated in L (250%) and LPG-amended soils (278%), both compared with control (Figure 6D).

### Carbon Metabolism

**FIGURE 7:**
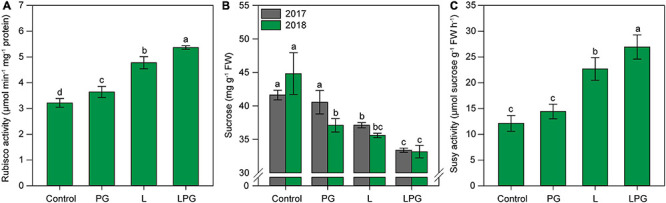
Rubisco activity **(A)**, sucrose concentration **(B)**, and Susy activity **(C)** in maize leaves as affected by surface-applied lime (L), phosphogypsum (PG), and lime + phosphogypsum (LPG) treatments. Different lower-case letters indicate significant differences between treatments for each growing season by Student’s *t*-test at *p* ≤ 0.05. Error bars express the standard error of the mean (*n* = 4).

The increased pigment concentrations in response to soil amendments were positively reflected in Rubisco activity (Figure 7A). Rubisco activity was highest (*p* < 0.01; Supplementary Table 6) in maize cultivated in LPG-amended soil (67% higher than control), followed by the L treatment (55% higher than control). Figure 7 The sucrose concentration was highest (*p* < 0.01; Supplementary Table 6) in the control treatment in both growing seasons (Figure 7B). In general, sucrose concentration in maize leaves decreased by 24% from control to the L and LPG treatments. The pattern of Susy activity was similar to that of Rubisco activity; Susy activity was highest (*p* < 0.01; Supplementary Table 6) in maize plants in the LPG-amended soil (120% higher than control), followed by the application of L alone (90% higher than control) (Figure 7C).

### Lipid Peroxidation and Antioxidant Metabolism

**FIGURE 8:**
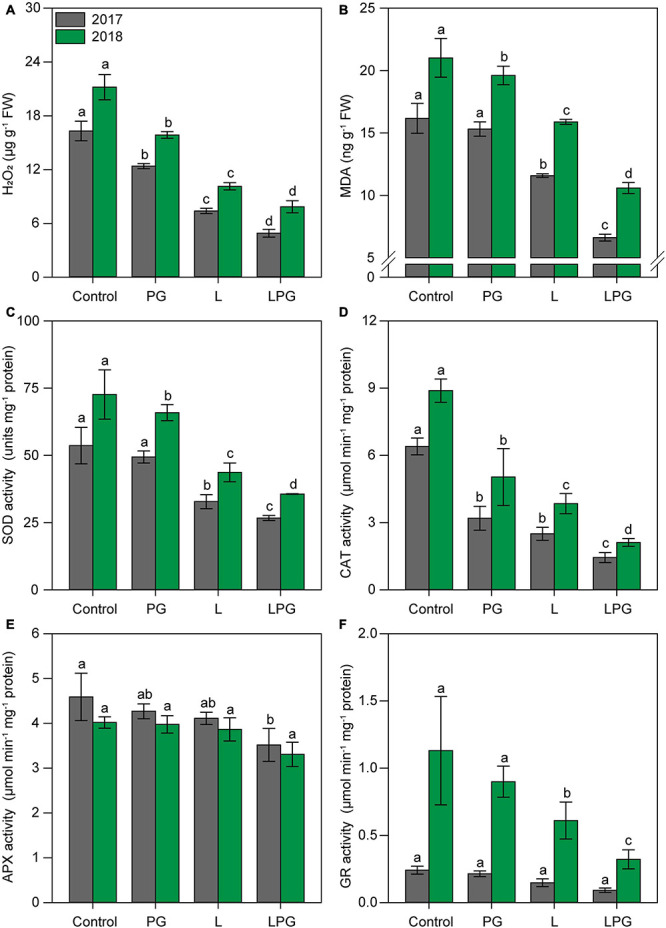
Oxidative stress [hydrogen peroxide (H_2_O_2_) **(A)** and malondialdehyde (MDA) **(B)** concentrations] and antioxidant enzyme activities [superoxide dismutase–SOD **(C)**, catalase–CAT **(D)**, ascorbate peroxidase–APX **(E)**, and glutathione reductase–GR **(F)**] in maize leaves as affected by surface-applied lime (L), phosphogypsum (PG), and lime + phosphogypsum (LPG) treatments. Different lower-case letters indicate significant differences between treatments for each growing season by Student’s *t*-test at *p* ≤ 0.05. Error bars express the standard error of the mean (*n* = 4).

Hydrogen peroxide (H_2_O_2_) and malondialdehyde (MDA) concentrations in maize leaves were highest (*p* < 0.01; Supplementary Table 6) in the control treatment (unamended soil) (Figures 8A,B). Regardless of growing season, the concentrations of H_2_O_2_ and MDA were reduced (*p* < 0.01; Supplementary Table 6) by all of the soil amendments compared with the control; however, these reductions were greatest in the LPG treatment (H_2_O_2_ = 66%; MDA = 54%), followed by the treatments with L (H_2_O_2_ = 53%; MDA = 13.7%) and PG (H_2_O_2_ = 24.6%; MDA = 6.4%) alone.

Antioxidant enzyme activities were highest (*p* < 0.01; Supplementary Table 6) in the leaves of maize in the control treatment or the treatment with PG alone (Figures 8C–F) in both growing seasons. On the other hand, enzymatic activity, i.e., SOD (Figure 8C), CAT (Figure 8D), APX (Figure 8E), and GR (Figure 8F) was lower (*p* < 0.01; Supplementary Table 6) in L-amended soil, especially in the LPG treatment. In general, considering both growing seasons, the antioxidant enzyme activities in maize cultivated in LPG-amended soil reduced by 51% for SOD, by 77% for CAT, by 21% for APX, and by 70% for GR, when compared with control treatment.

### Maize Shoot Dry Matter Production and Grain Yield

**FIGURE 9:**
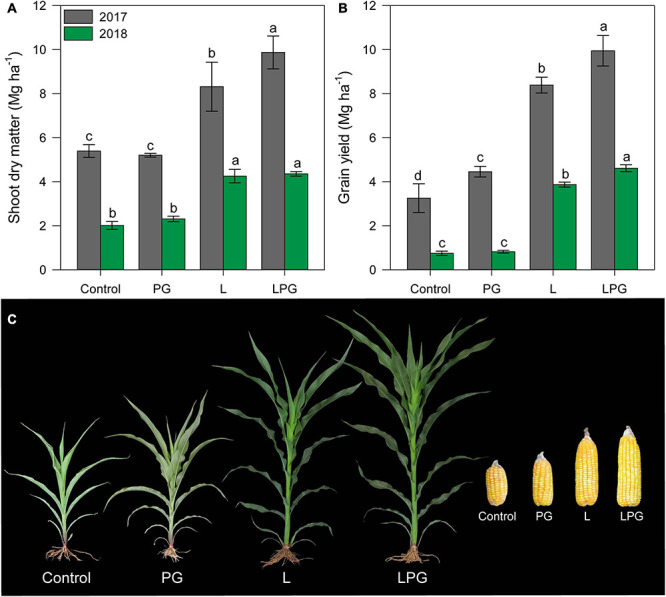
Shoot dry matter **(A)** and grain yield **(B)** of maize as affected by surface-applied lime (L), phosphogypsum (PG), and lime + phosphogypsum (LPG) treatments. Different lower-case letters indicate significant differences between treatments for each growing season by Student’s *t*-test at *p* ≤ 0.05. Error bars express the standard error of the mean (*n* = 4). Effect of soil amendments on maize development (2nd growing season = 2018) at 50 days after sowing and ear development at harvest **(C)**.

Figure 9 considering the average between the two growing seasons, SDM and GY were highest (*p* < 0.01; Supplementary Table 6) in the LPG treatment (SDM = 92%; GY = 260%), compared with control, followed by the L (SDM = 70%; GY = 200%), and PG (SDM = 1.35%; GY = 31%) (Figures 9A,B). A clear trend of LPG > L > PG > control was also observed for the development of maize plants at 50 days after emergence and ears obtained at harvest in the second growing season (Figure 9C), although some similarities were found between the L and LPG treatments and between the control and PG treatments.

### Redundancy and Correlation Analyses of Soil and Maize Plant Measurements

**FIGURE 10:**
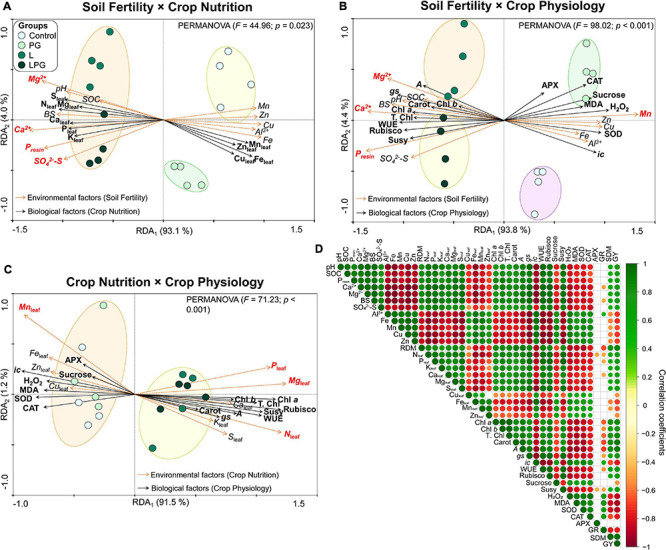
Redundancy analysis triplot (RDA) showing the relationship between the soil fertility × crop nutrition **(A)**, soil fertility × crop physiology **(B)**, and crop nutrition × crop physiology **(C)**. The canonical axes are labeled with percentage of total variance explained (%). The arrows indicate correlations between factors. The significance of these correlations was evaluated by a Monte Carlo permutation test with 999 permutations and the significant soil properties are indicated by red color (*p ≤* 0.05). The color dashed lines indicate significant clusters by permutation analysis (PERMANOVA, *p* ≤ 0.05). Heatmap showing the correlation coefficients (Pearson) among the soil fertility, root growth, crop nutrition, crop physiology, and agronomic parameters of maize plants **(D)**. Only significant correlations at *p* ≤ 0.05 are shown. Soil organic matter (SOC), base saturation (BS), sulfate (SO_4_^2–^-S), root dry matter (RDM), chlorophyll *a* (Chl *a*), Chlorophyll *b* (Chl *b*), total chlorophyll (T. Chl), carotenoids (Carot), Net photosynthesis rate (*A*), stomatal conductance (*gs*), internal CO_2_ concentration (*ic*), water use efficiency (WUE), hydrogen peroxide (H_2_O_2_), malondialdehyde (MDA), superoxide dismutase (SOD), catalase (CAT), ascorbate peroxidase (APX), glutathione reductase (GR), shoot dry matter (SDM), and grain yield (GY).

To elucidate the effects of soil fertility (considering the average of soil attributes at the 0.0–1.0 m depth) on crop nutrition and crop physiology (average of the two growing seasons), three RDAs were performed (Figures 10A–C). The first RDA contemplates the relationship between soil fertility and crop nutrition, where axis 1 indicated that the soil factors explained 93.1% of the variation in nutrient concentration in maize leaves (Figure 10A). PERMANOVA analysis segregated the treatments into the following three groups: group 1 represented by the control, group 2 composed by PG, and group 3 composed by L and LPG treatments. In addition, according to Monte Carlo permutation, the main soil factors (*p* < 0.05) responsible for all variation in crop nutrition were the soil concentrations of Ca^2+^, Mg^2+^, P_*resin*_, and SO_4_^2–^-S. Considering the role of soil fertility on crop physiology, PERMANOVA analysis segregated the treatments in four distinct groups, each represented by a treatment, with axis 1 indicating that soil fertility explained 93.8% of all variation in crop physiology (Figure 10B). The main soil factors (*p* < 0.05) responsible for these variations were the concentrations of Ca^2+^, Mg^2+^, P_*resin*_, and Mn. On the other hand, linking crop nutrition with maize physiology by RDA, axis 1 indicated that the crop nutrition was responsible for 91.5% of all variation in plant physiology (Figure 10C). In addition, PERMANOVA analysis segregated the treatments in two groups. Group 1 was composed by control and PG, whereas group 2 was composed by L and LPG treatments. Among the nutrients, Monte Carlo permutation indicated that the leaf concentrations of N, P, Mg, and Mn were the main responsible for changing (*p* < 0.05) the patterns of carbon and antioxidant metabolism in the maize. In general, Mn (soil or plant) has always been associated with an increase in oxidative stress, whereas the macronutrients (especially P and Mg) were associated with improved carbon metabolism.

Overall, correlation analysis of soil fertility and maize nutritional, physiological and productive attributes demonstrated that improvements in soil chemical properties enhanced the development and distribution of roots in the soil profile, resulting in higher concentration of nutrients in maize leaves, as well as improved the carbon fixation, lower oxidative stress and greater SDM, and GY (Figure 10D). Increased pH and the corresponding effects on soil properties (i.e., increased SOC and exchangeable cations and low Al^3+^ toxicity up to 1.0 m depth) were positively correlated with root growth, leaf macronutrient concentration, pigment concentrations, gas exchange parameters, Rubisco and Susy activities, and shoot and grain production. Additionally, increased pH was negatively correlated with leaf and soil micronutrient concentrations, lipid peroxidation, and the activities of SOD, CAT, APX, and GR.

## Discussion

### Climatic Conditions

In both growing seasons, the amounts of rainfall received during maize cropping were much lower than the range (400–600 mm) considered sufficient for crop development (Fancelli, 2015), particularly in the second growing season (105 mm less than 2017), resulting in a severely negative hydric balance during maize development (Figure 1). In both growing seasons, the hydric conditions for second-crop maize were inappropriate and negatively influenced crop development and yield. According to Gupta et al. (2020), drought is responsible for more annual losses of crop GY than all plant pathogens combined. Climatic anomalies related to longer periods of rain scarcity are becoming increasingly common in tropical regions (Cunningham, 2020; Heinemann et al., 2021), limiting food production. Tropical regions with weathered and acidic soils present low nutrient availability and high Al^3+^ levels, leading to restricted root growth and lower uptake of water and nutrients, especially under lower water supply (Ritchey et al., 1982; Joris et al., 2013).

### Changes in Soil Chemical Properties, Root Development, and Plant Nutrition

Remarkable effects of the soil amendments, primarily L, and LPG, were observed for most soil chemical properties, including higher soil pH, Ca^2+^, Mg^2+^, and SO_4_^2–^-S levels and lower levels of exchangeable Al^3+^ throughout the soil profile (Figure 1). As a result, there were significant correlations among soil pH, macronutrient availability and BS (Figure 10B). Additionally, in the 0.0–0.2 m layer, SOC content and P_*resin*_ concentration increased by the soil amendments application, although the increase in pH resulted in lower availability of cationic micronutrients (Figures 3, 8B). When PG was applied alone, soil attributes were slightly improved compared with the control treatment, but the effects were much smaller than those obtained by liming, regardless of the soil layer. However, when PG was applied in combination with L (LPG), the results were oftentimes greater than those obtained with L alone, especially in deeper soil layers. Unlike L, PG cannot correct soil acidity (Zoca and Penn, 2017), so its direct effects are linked to increased availability of Ca^2+^ and SO_4_^2–^-S and reduced Al^3+^ content, mainly in deep layers (Costa et al., 2018; Crusciol et al., 2019; Bossolani et al., 2021b). Since most soil properties co-vary with pH (Lammel et al., 2018), PG is an important complementary amendment but not a substitute for L (Bossolani et al., 2020a).

Changes in soil nutrient concentrations can alter root development in the soil profile (Rellán-Álvarez et al., 2016). Positive correlations with soil fertility occurred for root dry matter (Figure 10B). During plant development, the architecture of the root system undergoes morphological changes depending on the availability of water and nutrients in order to improve the acquisition of environmental resources (Carmeis Filho et al., 2017; Bossolani et al., 2021b). In tropical acidic soils, low Ca^2+^ availability and high Mn and Al^3+^ availability in deeper layers are the main causes of restricted root development. Calcium plays a fundamental role in root growth as a component of the hormonal peptides responsible for cell elongation (Ritchey et al., 1995), and when it is absent in deeper layers, roots become superficial, increasing the susceptibility of plants to drought stress and limiting nutrient uptake (Crusciol et al., 2019; Bossolani et al., 2021b). The root distribution in the soil profile can be traced to mechanisms of cell division, elongation, and differentiation mediated by nutrient availability (Rellán-Álvarez et al., 2016). Longer and deeper roots can efficiently take up water from deep layers (Gupta et al., 2020).

Nutrient uptake by maize increased in L- and LPG-amended soils (Supplementary Table 5). In general, the leaf concentration of all macronutrients was positively influenced by L (regardless of PG addition). However, the leaf concentration of cationic micronutrients decreased in these treatments in response to their reduced availability in soil (Figure 1), as supported by correlation analysis (Figure 10D). In addition, our RDA analysis regarding the interactions between soil fertility and crop nutrition revealed the primary effects higher soil concentrations of Ca^2+^, Mg^2+^, P_*resin*_, and SO_4_^2–^-S on improving the crop nutrition (Figure 10A). Thus, combining higher root development with increased soil nutrient concentrations, results on well-nourished plants that are able to withstand periods of low rainfall (Carmeis Filho et al., 2017; Bossolani et al., 2021b). In addition, according to Malavolta (1997), the macronutrient contents in maize were within the optimum range for full development only in soils amended with L or LPG, in both growing seasons.

### Photosynthetic Parameters and Carbon Metabolism Response

Amendment with L or LPG (Figures 1, 3) enabled greater root development and improved root distribution throughout the soil profile (Figure 2), as well as plant nutrition (Supplementary Table 5). Under low water availability like that observed in this study, plants seek to adapt to the soil moisture gradient and, driven by nutrient availability, change their root architecture to enhance their ability to take up water and nutrients (Rellán-Álvarez et al., 2016). These changes are reflected in greater synthesis of chlorophylls and carotenoids (Figure 5). Chlorophyll is an important part of the Calvin cycle and is responsible for harvesting sunlight during plant photosynthesis (Croft et al., 2017; Busch, 2020). Carotenoids are responsible for adapting plastids to stress conditions, light and energy dissipation to avoid excessive production of reactive oxygen species (ROS) (Gómez et al., 2019).

Correlation analysis suggested that the increase in photosynthetic pigment concentrations was related to changes in leaf nutritional status, gas exchange parameters and photosynthetic potential (Figure 10D). Rates of *A*, *gs*, and WUE were also increased by applying L alone or LPG (Figures 6A,B,D). In addition, the increases in these parameters in association with the decreases in *ic* (Figure 6C) and *E* (Figure 5) imply greater productive capacity of plants under stress conditions (Reis et al., 2018). The regulation of stomatal conductance under conditions of low hydric availability is extremely important for increased WUE (Gupta et al., 2020). According to these authors, stomatal closure is the first defense against water loss. Adequate plant nutrition (e.g., K, Ca, and Mg) are essential for the mechanisms that modulate this response (Brodribb and McAdam, 2017; Gong et al., 2020). According to our RDAs linking soil fertility (Figure 10B) and crop nutrition (Figure 10C) with crop physiology, *gs* values were strongly associated with the soil concentrations of exchangeable bases (Ca^2+^, Mg^2+^, and BS) and the leaf concentrations of K and Ca, reinforcing the role of these nutrients in improving the use of water under hydric restrictions, as occurred in our study. Additionally, greater availability of nutrients in the soil, especially in deeper layers, increases root growth and the use of resources that enhance resilience to low water availability (Rellán-Álvarez et al., 2016).

The combination of these factors may imply greater carbon fixation by plants (Reis et al., 2018). The key enzyme involved in carbon fixation that drives the assimilation of CO_2_ is Rubisco (Busch, 2020). In this study, Rubisco activity was higher in the leaves of maize in soils amended with L and, in particular, LPG (Figure 7A). Susy activity was also increased in these treatments (Figure 7C), although the levels of sucrose decreased (Figure 7B). The response pathways of plants to environmental stress also include changes in the production and mobilization of metabolites (Bailey-Serres et al., 2019). Each Rubisco-mediated carboxylation reaction gives rise to triose phosphates that are exported and become substrates for the synthesis of most of the other organic compounds that make up the plant (Stein and Granot, 2019). Sucrose synthase (Susy) plays a key role in sugar metabolism and can reversibly cleave sucrose into fructose and glucose. These by-products can enter several metabolic pathways to provide energy and carbon skeletons for plant metabolism (Stein and Granot, 2019). The increases in Rubisco and Susy activity and low sucrose concentrations in leaves suggest enhancement of production and partitioning of sugars from source to sink tissues (Farhat et al., 2016).

Redundancy analysis enabled the identification of the most important soil and nutritional factors responsible for increasing carbon metabolism (Figures 10B,C), that were also supported by correlation analysis (Figure 10D). Both RDAs presented a similar pattern correlating the soil and nutritional factors on carbon metabolism, however, the segregation of treatments was assembled differently. Soil fertility was more sensitive in clustering the treatments, segregating each treatment in a group; on the other hand, crop nutrition separated the treatments into only two groups: control and PG, and L and LPG treatments. Possibly, the segregation of treatments occurred differently considering soil fertility and crop nutrition due to the strong role of soil chemistry in modulating the soil nutrients availability, and the root growth, which directly contributes to the acquisition of water for the full functioning of photosynthesis (Rellán-Álvarez et al., 2016; Busch, 2020; Gong et al., 2020). Additionally, although the concentration of nutrients is an important indicator of the nutritional status of plants, it is worth noting that the leaf concentration of nutrients does not reflect the amount of nutrients that accumulate in the plant tissues. The foliar concentration of nutrients can be subject to the concentration and dilution processes, depending on the biomass production by plants (Maia, 2012). For this reason, the soil chemistry may have been more sensitive to changes that occurred in carbon metabolism. In general, soil and leaf P, Ca^2+^, and Mg^2+^ were the main nutrients responsible for improving the dynamics of Rubisco and Susy activities in maize plants, whereas Mn was mainly responsible for reductions in these activities, as observed in the control and PG treatments. Phosphorus is an important supplier of energy for photosynthetic mechanisms and plant metabolism. Mg^2+^ is the most abundant divalent cation in the plant cytosol and participates in several aspects of photosynthesis, such as the composition of the chlorophyll molecule, activation of Rubisco in the carboxylation process and the partitioning of photoassimilates by plant tissues (Ceylan et al., 2016). Calcium also plays an important role in multiple photosynthetic pathways (Wang et al., 2019). This nutrient affects gas exchange related to photosynthesis by regulating stomatal movement (Song et al., 2014). Several photosynthetic proteins are regulated directly or indirectly by Ca (Wang et al., 2019). In addition, soil Ca plays an important role in signaling and root growth, increasing the capacity of plants to uptake water and other nutrients (Ritchey et al., 1995).

In acidic and low-fertility soils, Mn is generally abundant and limits plant growth (Roth and Pavan, 1991; Bossolani et al., 2020b). Mn can replace Mg in the Rubisco activation process and increases the affinity of the enzyme for oxygen by approximately 20-fold compared with Mg^2+^, thus increasing photorespiration by plants (Kitao et al., 1997). In addition, high concentrations of metallic nutrients such as Mn and Al^3+^ can reduce the WUE of plants (Reis et al., 2018), compromising the crops in periods of low water availability. Several authors have also associated these elements with reduced root development (Roth and Pavan, 1991; Ritchey et al., 1995; Caires et al., 2011; Costa and Crusciol, 2016).

### Lipid Peroxidation and Antioxidant Metabolism Response

Oxidative stress in maize plants was strongly reduced by the application of L and LPG (Figures 8A,B), as evidenced by the reductions in the concentrations of H_2_O_2_ and MDA in these treatments. In addition to its role in cell defense signaling (Cuypers et al., 2010), H_2_O_2_ is one of the main forms of ROS responsible for lipid peroxidation (Loix et al., 2018).

The patterns of activity of antioxidant system enzymes (SOD, CAT, APX, and GR) (Figures 8C–F) were similar to those for lipid peroxidation, indicating that the low activity of these enzymes in maize established in L- and LPG-amended soils is the result of efficient catalysis of their substrates (ROS) (Silva et al., 2020). Lipid peroxidation can also be caused by other ROS [e.g., singlet oxygen (^1^O_2_^–^) and hydroxyl radicals (OH^–^)] (Farmer and Mueller, 2013). The first line of defense in antioxidant metabolism is the dismutation of ^1^O_2_^–^ into H_2_O_2_ and H_2_O by the enzyme SOD, followed by the breakdown of H_2_O_2_ into H_2_O and O_2_ by CAT, APX, and GR (Noctor, 2002). In this process of ROS scavenging, enzymes are also consumed, resulting in reduced activity when measured in plant tissue (Noctor, 2002; Farmer and Mueller, 2013).

The increased oxidative stress in maize plants in the control and PG treatments is primarily attributable to low soil fertility and plant nutrition, as supported by RDA and correlation analysis (Figures 10A–D). Plants grown under low nutrient availability, mainly P, Ca^2+^, and Mg^2+^, and under high concentrations of toxic elements, such as Mn and Al^3+^, are more likely to reduce pigment levels (Figure 5) and photosynthetic activity (Figures 5, 6; Liu et al., 2008, 2015; Gong et al., 2020), in addition to accumulating sugars in source tissues (Figure 7B; Stein and Granot, 2019). All of these factors are positively correlated with increased oxidative stress (Figure 10B; Gong et al., 2020). The gradual loss of photosynthetic capacity results in electron accumulation in photosystems I and II, generating greater amounts of ROS and lipid peroxidation (Croft et al., 2017). The accumulation of sucrose in photosynthetically active leaves interferes with the production of ROS and increases oxidative stress in plants, especially for plants growing under soil Mg^2+^ deficiency (Cakmak and Kirkby, 2008). Calcium deficiency induces low stomatal conductance (Figures 6B, 8B) and, consequently, higher water loss via stomata, which can increase oxidative stress in plants (Gupta et al., 2020), particularly under low hydric availability, which is common during maize cropping. In summary, the mitigation of soil acidification, reduced toxicity of Mn and Al^3+^, and increased availability of nutrients such as P, Ca^2+^, and Mg^2+^ provided by liming (L), especially when combined with PG (LPG), can reduce oxidative stress in plants.

### Maize Shoot Dry Matter Production and Grain Yield

This study revealed positive effects of the soil amendments on root development, plant carbon and antioxidant metabolism, SDM production and GY (Figure 9). In addition, during maize development, more turgid and vigorous plants were obtained, even under low pluvial precipitation (Figure 1). The long-term application of L and LPG improved soil fertility and root development, increasing the water and nutrients uptake by plants (Table 1). In addition, increased photosynthesis and better regulation of oxidative stress led to higher maize GYs under these soil amendments. Importantly, combining L and PG (LPG) provided better results than amendment with L alone. Understanding how soil amendments charges tropical soils and shape plant responses under drought is of paramount importance for the development of more productive agricultural systems under continuous climate change (Gibbons et al., 2014; Gupta et al., 2020). Therefore, amendment of soils with LPG should be considered a viable and promising alternative to improve the yield capacity of acidic tropical soils managed under NT (Costa and Crusciol, 2016; Crusciol et al., 2019; Bossolani et al., 2020a, b, 2021b).

## Conclusion

After 2 years of the last surface reapplication of soil amendments (four reapplications of lime and/or phosphogypsum in 16 years of experiment), liming, especially combined with phosphogypsum, increased soil pH, P, Ca^2+^, Mg^2+^, and SO_4_^2–^-S and reduced the concentrations of Al^3+^, Fe, Mn, Cu, and Zn beyond the depth where lime was applied. The improvement on soil fertility brought about by soil amendments, led to an increase on root growth of maize, which in turn, increased water and nutrient uptake by plants. As a result of this chain effect, maize plants grown under field drought conditions, improved their antioxidant system, photosynthetic pigment concentrations and, consequently, the carbon metabolism, including Rubisco and Susy activities, and sucrose production and partitioning. This study has not only confirmed that phosphogypsum potentiates the effects of lime on improving soil fertility, but has also highlighted that the increase in macronutrient concentrations (in soil and plants), especially P, Ca, and Mg, and reducing Mn concentrations, has a fundamental role in increasing antioxidant system and photosynthetic metabolism, benefits that eventually were reflected in the increase of the biomass production and GY.

## Data Availability Statement

The raw data supporting the conclusions of this article will be made available by the authors, without undue reservation.

## Author Contributions

JB and CC worked on the research designing and conduction, data analysis, and writing and formatting the manuscript. AG, LM, JP, VR, MF, JC, EC, TA, and AR revised this draft by rewriting, discussing, and commenting. All authors contributed significantly on the manuscript, confirm being contributor of this work, and approved it for publication.

## Conflict of Interest

The authors declare that the research was conducted in the absence of any commercial or financial relationships that could be construed as a potential conflict of interest.

## Footnotes

1 https://www.glten.org/experiments/62

2 www.calcarioguapirama.com.br
