# Supplementary material for: Long-Term Lime and Phosphogypsum Amended-Soils Alleviates the Field Drought Effects on Carbon and Antioxidative Metabolism of Maize by Improving Soil Fertility and Root Growth
Source: Front Plant Sci. 2021 Jul 12;12:650296. doi: 10.3389/fpls.2021.650296 (PMC8313040; doi:10.3389/fpls.2021.650296)
Supplement: Supplementary file 1 [file Data_Sheet_1.docx]

**SUPPLEMENTARY MATERIAL**

**Long-term lime and phosphogypsum amended-soils alleviates the field drought effects on carbon and antioxidative metabolism of maize by improving soil fertility and root growth**

João William Bossolani^a^, Carlos Alexandre Costa Crusciol^a*^, Ariani Garcia^a^, Luiz Gustavo Moretti^a^, José Roberto Portugal^a^, Vitor Alves Rodrigues^a^, Mariley de Cássia da Fonseca^a^, Juliano Carlos Calonego^a^, Eduardo Fávero Caires^b^, Telmo Jorge Carneiro Amado^c^, André Rodrigues dos Reis^d^

^a^ São Paulo State University (UNESP), College of Agricultural Sciences, Department of Crop Science, 18610-034, Botucatu, São Paulo, Brazil.

^b^ State University of Ponta Grossa (UEPG), Department of Soil Science and Agricultural Engineering, 84030-900, Ponta Grossa, Paraná, Brazil

^c^ Federal University of Santa Maria (UFSM), Center of Rural Sciences (CRS), Soils Department, 97105-900, Santa Maria, Rio Grande do Sul, Brazil

^d^ São Paulo State University (UNESP), School of Sciences and Engineering, Department of Biosystems Engineering, 17602-496, Tupã, São Paulo, Brazil

**^*^Corresponding author:**

São Paulo State University (UNESP), College of Agricultural Sciences, Department of Crop Science, 18610-034, Botucatu, São Paulo, Brazil.

E-mail address: carlos.crusciol@unesp.br (C.A.C. Crusciol).

**Table S1.** Crops growing and treatments application scheme during the experimental period (from 2002 to 2018).

| Growing season | Seasons | | Treatments^†^ |
| --- | --- | --- | --- |
|  | Summer crops | Autumn-Winter-Spring crops |  |
| 2002/2003 | *Oriza sativa* | *Avena strigosa* | Lime: 2.7 Mg ha^-1^  (71% ECCE^‡^)  Phosphogypsum: 2.1 Mg ha^-1^ |
| 2003/2004 | *Phaseolus vulgaris* | *Avena strigosa* | - |
| 2004/2005 | *Arachis hypogaea* | *Avena sativa* | Lime: 2.0 Mg ha^-1^  (71% ECCE)  Phosphogypsum: 2.1 Mg ha^-1^ |
| 2005/2006 | *Arachis hypogaea* | *Avena sativa* | - |
| 2006/2007 | *Zea mays* intercropped with *Urochloa brizantha* (cv. Marandu) | *Urochloa brizantha* (cv. Marandu)  (after *Zea mays* harvest) | - |
| 2007/2008 | *Zea mays* intercropped with *Urochloa brizantha* (cv. Marandu) | *Urochloa brizantha* (cv. Marandu)  (after *Zea mays* harvest) | - |
| 2008/2009 | *Glycine max* | *Avena strigosa* | - |
| 2009/2010 | *Glycine max* | *Sorghum vulgare* | - |
| 2010/2011 | *Zea mays* | *Crambe abyssinica / Vigna unguiculata* | Lime: 2.0 Mg ha^-1^  (88% ECCE)  Phosphogypsum: 2.1 Mg ha^-1^ |
| 2011/2012 | *Zea mays* | *Crambe abyssinica / Vigna unguiculata* | - |
| 2012/2013 | *Pennisetum glaucum* | *Triticum aestivum* | - |
| 2013/2014 | *Phaseolus vulgaris* | *Triticum aestivum* | - |
| 2014/2015 | *Phaseolus vulgaris* | *Urochloa brizantha* (cv. Marandu)  (1^st^ growing season) | - |
| 2015/2016 | *Urochloa brizantha* (cv. Marandu)  (2^nd^ growing season) | *Urochloa brizantha* (cv. Marandu)  (3^rd^ growing season) | - |
| 2016/2017^§^ | *Glycine max* | *Zea mays* intercropped with *Urochloa ruziziensis* | Lime: 13 Mg ha^-1^ (69% ECCE)  Phosphogypsum: 10 Mg ha^-1^ |
| 2017/2018 | *Glycine max* | *Zea mays* intercropped with *Urochloa ruziziensis* | - |

^†^The reapplications in October 2004, 2010 and 2016 were performed when base saturation reached ≤ 50%.

^‡^ECCE: effective calcium carbonate equivalent

^§^Micronutrients applied in total area

**Table S2.** Statistical parameters by ANOVA and Student's t-test at *p* ≤ 0.05 significance level of experimental factors [control (no soil amendment application), lime (L), phosphogypsum (PG), and lime + phosphogypsum (LPG)] for soil chemical properties in stratified layers (0.0–1.0 m depth).

| Soil properties | Soil depth (m) | | | | | |
| --- | --- | --- | --- | --- | --- | --- |
|  | 0.0–0.1 | 0.1–0.2 | 0.2–0.4 | 0.4–0.6 | 0.6–0.8 | 0.8–1.0 |
| pH | <0.001 | <0.001 | 0.0271 | <0.001 | 0.0016 | 0.0048 |
| LSD^†^ | 0.27 | 0.33 | 0.48 | 0.14 | 0.18 | 0.16 |
| CV^‡^ (%) | 3.32 | 4.51 | 6.98 | 2.10 | 2.76 | 2.59 |
| Ca^2+^ | <0.001 | <0.001 | <0.001 | <0.001 | <0.001 | <0.001 |
| LSD | 2.96 | 2.25 | 2.13 | 1.08 | 1.15 | 0.50 |
| CV (%) | 4.20 | 5.22 | 5.87 | 5.01 | 9.12 | 5.80 |
| Mg^2+^ | <0.001 | <0.001 | <0.001 | <0.001 | <0.001 | <0.001 |
| LSD | 1.88 | 1.34 | 1.87 | 1.86 | 1.72 | 0.49 |
| CV (%) | 6.59 | 7.64 | 14.4 | 15.3 | 16.5 | 15.4 |
| BS | <0.001 | <0.001 | <0.001 | <0.001 | <0.001 | <0.001 |
| LSD | 3.62 | 5.07 | 4.73 | 2.07 | 1.53 | 0.55 |
| CV (%) | 4.32 | 7.71 | 9.98 | 6.20 | 6.94 | 4.16 |
| Al^3+^ | <0.001 | <0.001 | <0.001 | <0.001 | <0.001 | <0.001 |
| LSD | 1.16 | 5.94 | 1.61 | 3.18 | 3.83 | 3.97 |
| CV (%) | 15.3 | 29.4 | 11.7 | 16.2 | 16.0 | 16.2 |
| SO_4_^2-^-S | <0.001 | <0.001 | <0.001 | <0.001 | <0.001 | <0.001 |
| LSD | 3.68 | 5.42 | 7.74 | 7.77 | 7.58 | 10.6 |
| CV (%) | 10.6 | 12.0 | 12.5 | 11.0 | 9.87 | 13.7 |

Exchangeable calcium (Ca^2+^), magnesium (Mg^2+^) and aluminum (Al^3+^), base saturation (BS) and sulfate (SO_4_^2-^-S). ^†^Low significant difference (LSD), ^‡^coefficient of variation (CV)

**Table S3.** Statistical parameters by ANOVA and Student's t-test at *p* ≤ 0.05 significance level of experimental factors [control (no soil amendment application), lime (L), phosphogypsum (PG), and lime + phosphogypsum (LPG)] for soil chemical properties at 0.0–0.2 m depth.

| Treatments | SOM | P | Fe | Mn | Cu | Zn |
| --- | --- | --- | --- | --- | --- | --- |
| *p value* | <0.001 | <0.001 | <0.001 | <0.001 | 0.2023 | 0.0161 |
| LSD^†^ | 1.59 | 4.14 | 3.16 | 5.05 | 0.50 | 0.76 |
| CV^‡^ (%) | 3.61 | 7.44 | 8.61 | 9.64 | 15.8 | 14.7 |

Soil organic matter (SOM), phosphorus (P), iron (Fe), manganese (Mn), copper (Cu) and zinc (Zn)

^†^Low significant difference (LSD), ^‡^coefficient of variation (CV)

**Table S4.** Statistical parameters by ANOVA and Student's t-test at *p* ≤ 0.05 significance level of experimental factors [control (no soil amendment application), lime (L), phosphogypsum (PG), and lime + phosphogypsum (LPG) × two growing seasons] for maize root dry matter and root dry matter distribution in stratified layers (0.0–1.0 m depth).

| Treatments | Soil depth (m) | | | | | |
| --- | --- | --- | --- | --- | --- | --- |
|  | 0.0–0.1 | 0.1–0.2 | 0.2–0.4 | 0.4–0.6 | 0.6–0.8 | 0.8–1.0 |
|  | Root dry matter | | | | | |
| SA* (2017) | <0.001 | <0.001 | <0.001 | <0.001 | <0.001 | <0.001 |
| LSD^†^ | 0.0652 | 0.0570 | 0.0482 | 0.0364 | 0.0338 | 0.0267 |
| SA (2018) | <0.001 | <0.001 | <0.001 | <0.001 | <0.001 | <0.001 |
| LSD | 0.0539 | 0.0506 | 0.0423 | 0.0320 | 0.0237 | 0.0205 |
| CV^‡^ (%; 2017) | 8.54 | 7.55 | 9.75 | 10.90 | 9.04 | 8.39 |
| CV^‡^ (%; 2018) | 6.03 | 5.73 | 7.13 | 7.83 | 6.53 | 7.23 |
|  | Root dry matter distribution | | | | | |
| SA (2017) | <0.001 | <0.001 | 0.3454 | <0.001 | <0.001 | <0.001 |
| LSD | 2.28 | 1.73 | 1.35 | 1.49 | 1.37 | 0.96 |
| SA (2018) | <0.001 | <0.001 | 0.4298 | <0.001 | <0.001 | <0.001 |
| LSD | 3.84 | 2.96 | 1.43 | 1.13 | 1.09 | 0.75 |
| CV^‡^ (%; 2017) | 4.27 | 3.17 | 7.68 | 5.58 | 9.37 | 9.67 |
| CV^‡^ (%; 2018) | 6.49 | 3.89 | 8.78 | 7.36 | 11.37 | 8.43 |

*Soil amendments (SA), ^†^Low significant difference (LSD), ^‡^coefficient of variation (CV)

**Table S5.** Statistical parameters by ANOVA and Student's t-test at *p* ≤ 0.05 significance level of experimental factors [control (no soil amendment application), lime (L), phosphogypsum (PG), and lime + phosphogypsum (LPG) × two growing seasons] for nutritional status of maize.

| Treatments | N | P | K | Ca | Mg | S | Fe | Mn | Cu | Zn |
| --- | --- | --- | --- | --- | --- | --- | --- | --- | --- | --- |
| SA* (2017) | <0.001 | 0.1267 | 0.6117 | 0.0037 | <0.001 | <0.001 | 0.0236 | 0.0094 | 0.6967 | <0.001 |
| LSD^†^ | 3.87 | 0.165 | 2.78 | 1.09 | 1.26 | 0.49 | 74.1 | 5.08 | 4.58 | 17.7 |
| SA (2018) | <0.001 | 0.0235 | 0.041 | 0.1266 | <0.001 | <0.001 | 0.0016 | 0.0120 | 0.1156 | 0.0018 |
| LSD | 3.90 | 0.23 | 2.60 | 0.59 | 1.14 | 0.59 | 41.5 | 5.08 | 2.91 | 16.2 |
| CV^‡^ (%; 2017) | 7.15 | 4.43 | 8.35 | 21.4 | 20.4 | 11.6 | 21.0 | 13.1 | 22.6 | 17.8 |
| CV (%; 2018) | 8.55 | 7.02 | 9.00 | 14.8 | 20.5 | 18.2 | 13.0 | 13.9 | 15.4 | 19.3 |

*Soil amendments (SA), Nitrogen (N), phosphorus (P), potassium (K), calcium (Ca), magnesium (Mg), sulfur (S), iron (Fe), manganese (Mn), copper (Cu) and zinc (Zn). ^†^Low significant difference (LSD), ^‡^coefficient of variation (CV)

.

**Table S6.** Statistical parameters by ANOVA and Student's t-test at *p* ≤ 0.05 significance level of experimental factors [control (no soil amendment application), lime (L), phosphogypsum (PG), and lime + phosphogypsum (LPG) × two growing seasons] for maize physiological, biochemical, and agronomic parameters.

| Treatments | Chl *a* | Chl *b* | Total chl | Carot | *A* | *gs* | *E* | *ic* | WUE | Rubisco |
| --- | --- | --- | --- | --- | --- | --- | --- | --- | --- | --- |
| SA* (2017) | <0.001 | <0.001 | <0.001 | <0.001 | <0.001 | <0.001 | 0.0011 | <0.001 | <0.001 | - |
| LSD | 4.21 | 0.93 | 5.13 | 0.66 | 2.46 | 0.48 | 1.64 | 8.38 | 1.23 | - |
| SA (2018) | <0.001 | <0.001 | <0.001 | <0.001 | <0.001 | <0.001 | <0.001 | <0.001 | <0.001 | <0.001 |
| LSD | 3.15 | 0.77 | 3.93 | 0.51 | 1.80 | 0.49 | 0.40 | 9.51 | 0.95 | 0.32 |
| CV^‡^ (%; 2017) | 20.1 | 20.6 | 20.2 | 10.3 | 5.25 | 11.0 | 19.4 | 7.26 | 11.8 | - |
| CV (%; 2018) | 20.7 | 20.8 | 20.7 | 10.5 | 5.35 | 12.6 | 5.49 | 7.34 | 9.73 | 4.76 |
|  |  |  |  |  |  |  |  |  |  |  |
|  | Sucrose | Susy | H_2_O_2_ | MDA | SOD | CAT | GR | APX | SDM | GY |
| SA (2017) | <0.001 | - | <0.001 | <0.001 | <0.001 | <0.001 | <0.001 | 0.0176 | <0.001 | <0.001 |
| LSD | 1.46 | - | 0.98 | 1.21 | 5.79 | 0.63 | 0.04 | 0.60 | 1.14 | 0.79 |
| SA (2018) | 0.0156 | <0.001 | <0.001 | <0.001 | <0.001 | <0.001 | 0.0023 | 0.0011 | <0.001 | <0.001 |
| LSD | 2.34 | 2.64 | 1.30 | 1.59 | 7.94 | 1.23 | 0.34 | 0.29 | 0.30 | 0.21 |
| CV^‡^ (%; 2017) | 2.38 | - | 5.99 | 6.09 | 8.91 | 11.6 | 15.1 | 9.06 | 9.90 | 7.55 |
| CV (%; 2018) | 3.88 | 8.66 | 5.91 | 5.92 | 9.12 | 15.5 | 28.5 | 4.71 | 5.85 | 5.11 |

*Soil amendments (SA), Chlorophyll *a* (Chl *a*), Chlorophyll *b* (Chl *b*), total chlorophyll (Total chl), carotenoids (Carot), Net photosynthesis rate (*A*), stomatal conductance (*gs*), internal CO_2_ concentration (*ic*), transpiration (*E*), water use efficiency (WUE), hydrogen peroxide (H_2_O_2_), malondialdehyde (MDA), superoxide dismutase (SOD), catalase (CAT), ascorbate peroxidase (APX) and glutathione reductase (GR), shoot dry matter (SDM) and grain yield (GY). ^†^Low significant difference (LSD), ^‡^coefficient of variation (CV).


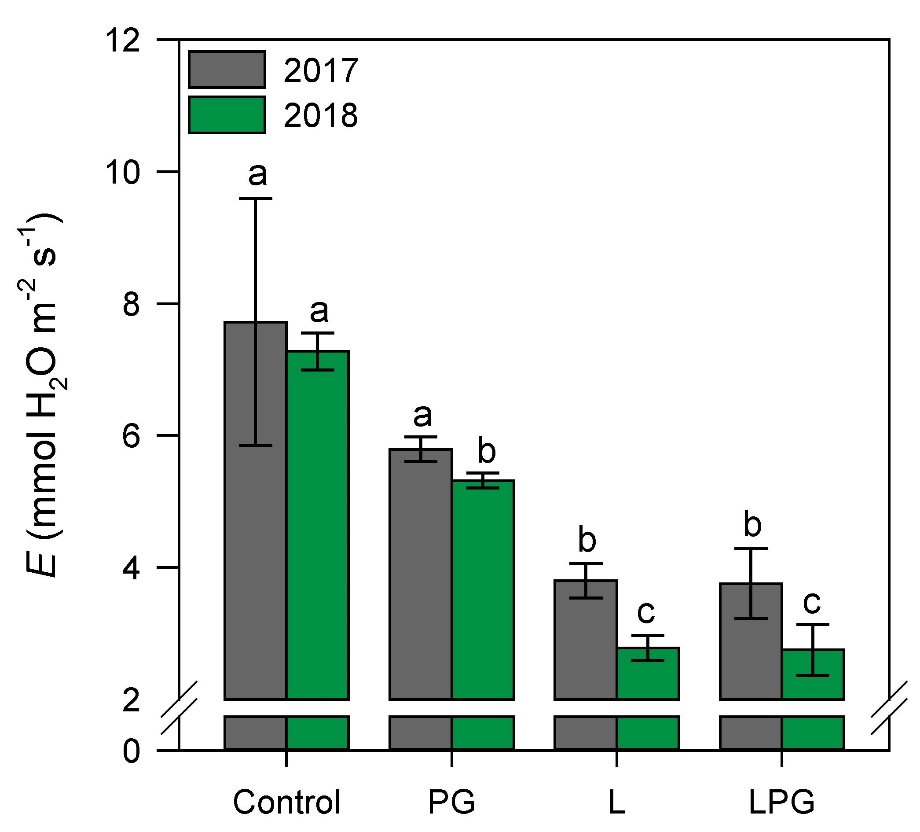


**Figure S1.** Leaf transpiration rate (*E*) in maize leaves as affected by surface-applied lime (L), phosphogypsum (PG), and lime + phosphogypsum (LPG) treatments. Different lower-case letters indicate significant differences between treatments for each growing season by Student's t-test at *p* ≤ 0.05. Error bars express the standard error of the mean (*n* = 4).
